# Supplementary material for: Sensitivity of the Natriuretic Peptide/cGMP System to Hyperammonaemia in Rat C6 Glioma Cells and GPNT Brain Endothelial Cells
Source: Cells. 2021 Feb 15;10(2):398. doi: 10.3390/cells10020398 (PMC7919485; doi:10.3390/cells10020398)
Supplement: Supplementary file 1 [file cells-10-00398-s001.pdf]

Supplemental Table S1 Primer sequences for multiplex RT-qPCR assays

Gene name	Accession Number	Product Size	Left Sequence	Right Sequence
Abcc4	NM_133411	100	CAGGATGCCGACATCTACCT	CGTGCAAAGTCTGGCAGATA
ActB/B-Actin	NM_007393	107	GTACCACCATGTACCCAGGC	GTACTTGCGCTCAGGAGGAG
Pde7a	NM_031080	114	GCTCAGTTTCCAGCGGTATC	TCCAGCATACACTTGGCTTG
Prkg1	NM_001105731	128	AATCCGAGAGGTCGAAGGAT	GCTGTCCTTCCCATATTCCA
Pde11a	NM_001127481	135	GATTCTGACCGAGGTGGAAA	GTCGCTGAGGTCCCATAGAG
Pde8b	NM_199268	142	CATGTTTGATGCTTGGGATG	TCTGGCTTGGCTTTAGCTGT
Pde4b	NM_017031	149	CAAGCAGAACGATGTGGAAA	TGACTCCAAAGCGTGAGATG
Pde9a	NM_138543	156	GGTGGAATTGGAAGGACTCA	ACATCACGTCGAGGTGTCAA
Rpl19	NM_009078	163	GAGCACATCCACAAGCTGAA	TCTCCTCCTCCTTGGACAGA
Pde4d	NM_001113329	170	GGACCGGATAATGGAGGAGT	GATGTACGAGGTCTGCCCAT
Pde4a	NM_013101	177	TTCATCCATTGTGGGAGACA	ATTGGAACTTGTCAGGCAGG
Prkg2	NM_013012	184	CAAGCCAGGGATGAGGAATA	TCACCTTTCCTTTTGCCAAG
Pde1a	NM_030871	193	AAGCAGCTGGAGAAAGGTGA	TCCGTGTAAAGGTTGAAGCC
Pde10a	NM_022236	200	GGTCTTGTGGGACATCCTGT	GCACAGACAGGCAATTAGCA
Abcc5	NM_053924	207	CCCAACAGGAAGGATTCTCA	AGGACCCTGGAGACAATGTG
Pde5a	NM_133584	214	AAGGATTCTGAGGGAACCGT	GGGAATAGCGATCAGCAGAG
Npr1	NM_008727	121	CTTGGAATTCCTGAAGCAGC	CTGGACATAGAGCAGGAGCC
Npr2	NM_173788	149	CCTTGATGTCTTTGGGGAGA	GATTTGGGGGTTCTCGGTAT
Gfap	NM_017009	156	GAAGAAAACCGCATCACCAT	CGACTCCTTAATGACCTCGC
Npr3	NM_008728	198	TCTGCTGTCCTCTGTCCCTT	CTGGTTTTGAAGGGCATCAT
Hmox1	NM_012580	100	ACCCCACCAAGTTCAAACAG	AAGGCGGTCTTAGCCTCTTC
Gad1	NM_017007	114	GTGCAGGCTACCTCTTCCAG	TCCACATCAGCCAGAACTTG
Gad2	NM_012563	136	CCAGCCTGTGAAGGAGAAAG	CTTGAAGAAGCTCATTGGGG
Fos	NM_022197	197	GGGAGCTGACAGATACGCTC	AAAGTCCAGGGAGGTCACAGA
Kan(r)	n/a	288	ATCATCAGCATTGCATTCGATTCCTGTTTG	ATTCCGACTCGTCCAACATC
